# Supplementary material for: A multimodal iPSC platform for cystic fibrosis drug testing
Source: Nat Commun. 2022 Jul 29;13:4270. doi: 10.1038/s41467-022-31854-8 (PMC9338271; doi:10.1038/s41467-022-31854-8)
Supplement: Supplementary file 1 — Supplementary Information [file 41467_2022_31854_MOESM1_ESM.pdf]

A multimodal iPSC platform for cystic fibrosis drug testing

Supplementary Information

Supplementary Figure 1

Code	Cell Line	CFTR Genotype	Cell Source	Karyotype		Notes
non-CF #1	RUES2	Normal	Embryonic	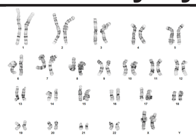	46 XX	The Rockefeller University
non-CF #2	BU1	Normal	Dermal Fibroblasts	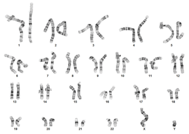	46 XY	Park et al, 2016 Mithal et al, 2020
non-CF #3	BU3	Normal	PBMCs	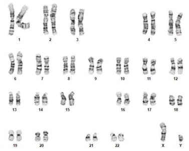	46 XY	Hawkins et al, 2021
W1282X #1	P20801	W1282X/ W1282X	PBMCs	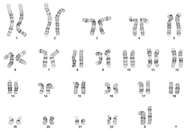	46 XX	University of Pennsylvania (Diamond Lab)
W1282X #2	TorontoCF2	W1282X/ W1282X	PBMCs	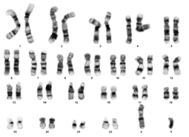	46 XY	University of Toronto Hospital for Sick Children
G542X	C17*	G542X/ G542X	Dermal Fibroblasts	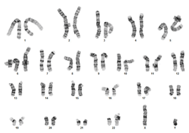	46 XY	Cystic Fibrosis Foundation
G551D #1	CFTR5-5	G551D/ G551D	PBMCs	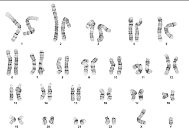	46 XY	Children's Health Ireland
G551D #2	C17*	G551D/ G551D	Dermal Fibroblasts	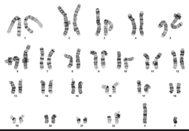	46 XY	Cystic Fibrosis Foundation
G551D #3	TorontoCF1	G551D/ G551D	PBMCs	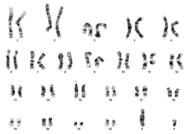	46 XY	University of Toronto Hospital for Sick Children
Phe508del #1	CFTR4-2	Phe508del/ Phe508del	PBMCs	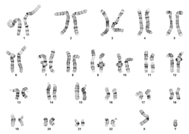	46 XY	Children's Health Ireland
Phe508del #2	RC2 204	Phe508del/ Phe508del	Dermal Fibroblasts	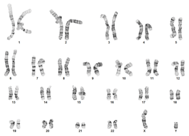	46 XY	Somers et al, 2010
Phe508del #3	C17	Phe508del/ Ile507del	Dermal Fibroblasts	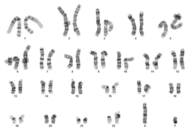	46 XY	Crane et al, 2015

*Gene-editing to corresponding CFTR variants noted

Supplementary Figure 1. Description and characterization of pluripotent stem cell lines. Cell lines shown with de-identified coding, as well as original cell source and *CFTR* genotype. Karyotypes were confirmed as normal for all cell lines and markers of pluripotency were assessed with immunofluorescent staining or flow cytometry as indicated. Scale bars represent 500μm.

Supplementary Figure 2

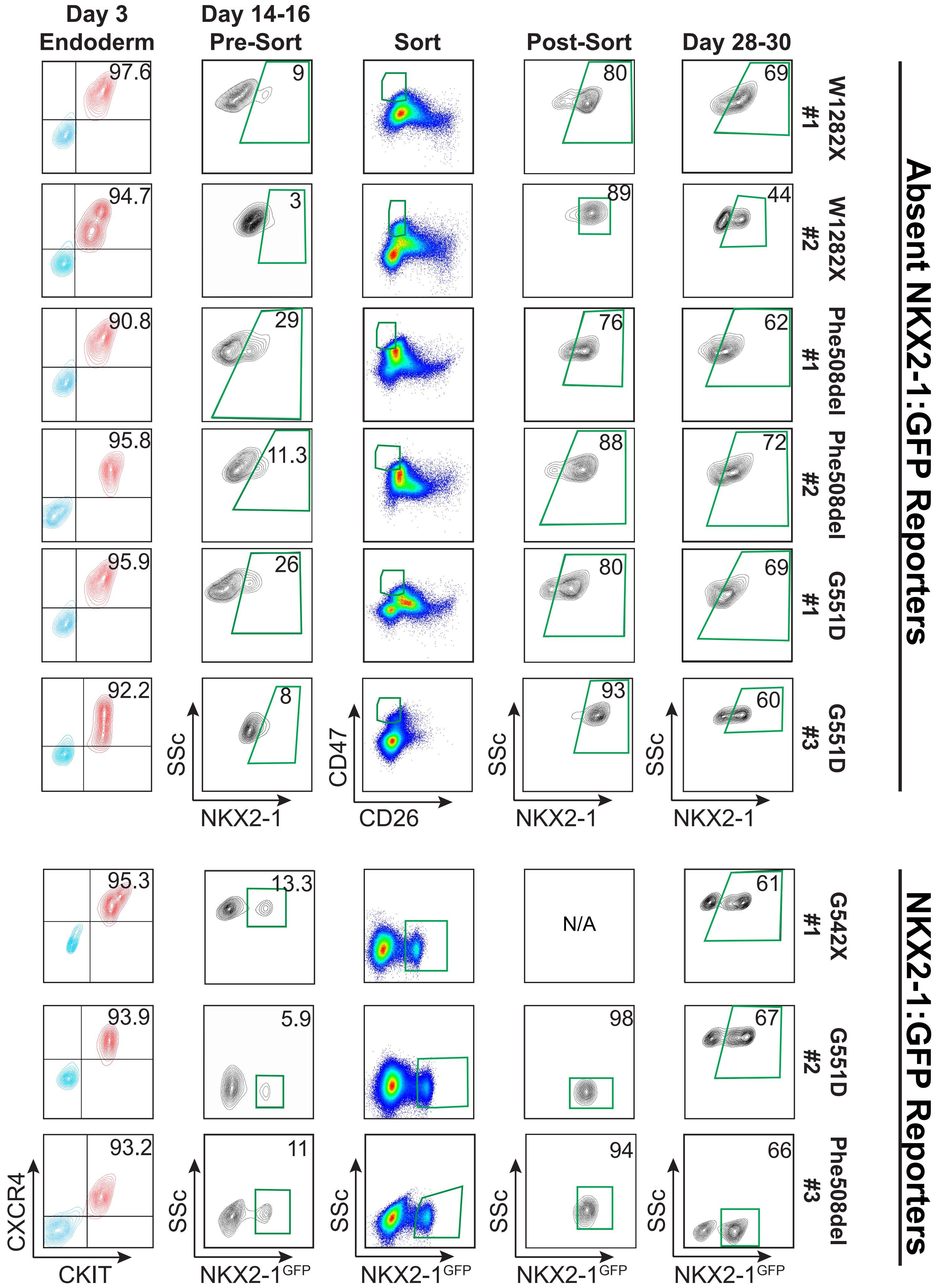

Supplementary Figure 2. Flow cytometry assessment of lung-directed differentiation checkpoints for CF cell lines. Each cell line, shown in a separate row, was analyzed at the timepoints shown to ensure adequate progress of directed differentiation. Day 3 samples (column 1) were assessed by co-expression of cell surface markers CKIT and CXCR4; stained samples shown in red, isotype controls shown in blue. Samples were assessed on day 14-16 (prior to sorting), immediately post-sorting, and on day 28-30 for intracellular NKX2-1 expression as shown (columns 2, 4, 5). Samples were enriched for NKX2-1 on day 14-16 using the $CD47^{hi}/CD26^{neg}$ cell sorting strategy, shown in column 3. Flow cytometry and sorting of Phe508del #3 (row 5) utilized an NKX2-1:GFP fluorescent reporter, rather than intracellular NKX2-1 staining or CD47hi/CD26neg sorting, respectively. Numbers overlaid onto flow cytometry plots represent the percentage of cells for the gate shown. Gating was determined using known negative cells, of a similar differentiation day.

Supplementary Figure 3

A)

	Cell Line	Average/Experiment	Average/Condition	Experiments
Non-CF	non-CF #1	379	95	3
	non-CF #2	219	110	3
	non-CF #3	484	242	3
CF	W1282X #1	427	107	3
	W1282X #2	946	158	3
	G542X	1253	157	3
	Phe508del #1	557	139	8
	Phe508del #2	598	120	9
	Phe508del #3	1170	234	9
	G551D #1	344	86	5
	G551D #2	189	63	3
	G551D #3	543	181	3
Means	Overall	592	137	
	Non-CF	361	149	
	CF	685	133	

		Per Exp't	Per Condition
Overall	AVERAGE	592	141
	SEM	101	16
Non-CF	AVERAGE	361	149
	SEM	77	47
CF	AVERAGE	670	138
	SEM	214	17

B)

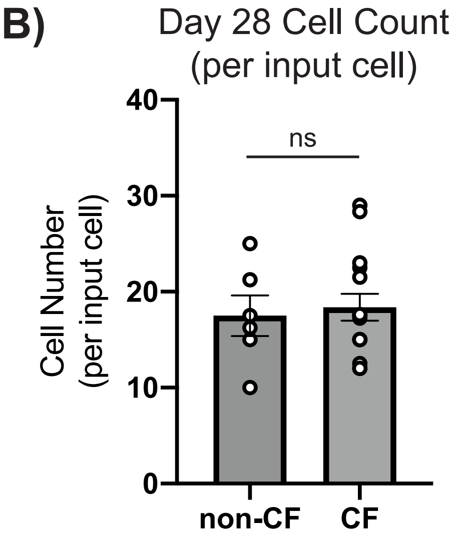

C)

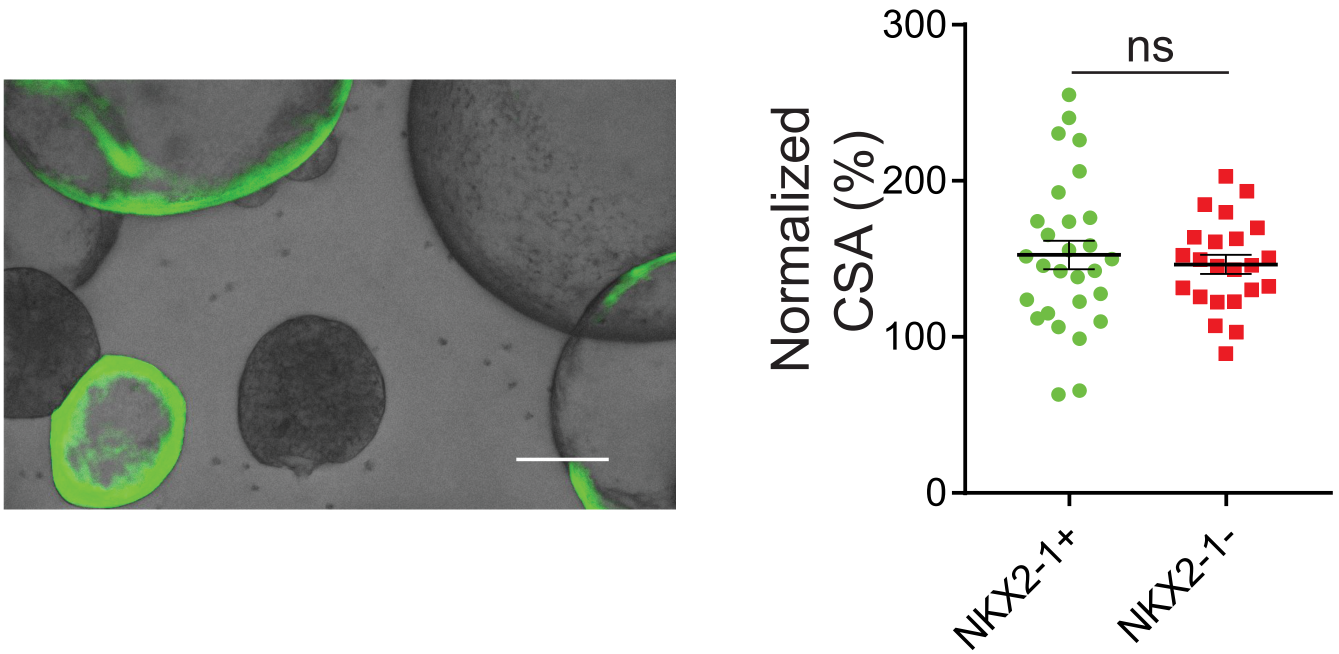

Supplementary Figure 3. Quantification of spheroid numbers and magnitude of NKX2-1+ versus NKX2-1- spheroid FIS. A) Spheroid numbers shown represent the number analyzed in FIS assay (note: spheroids on the edge of imaging or under the size threshold were not counted). **B)** CF and non-CF spheroids contain similar number of cells. Each point represents an individual experiment (independent biological experiments: n=6 for non-CF, n=12 for CF). **C)** Spheroids from non-CF #3 were generated and imaged using live cell fluorescence microscopy. Utilizing the NKX2-1:GFP fluorescent reporter, we identifed NKX2-1^{GFP+} and NKX2-1^{GFP-} spheroids prior to FIS (left panel). After 24 hours of forskolin stimulation, spheroid change in CSA is shown; each point represents an individual spheroid (n=28 for NKX2-1+, n=24 for NKX2-1-). Lines and error bars represent mean and standard error; unpaired two-tailed Student's t-test was performed for statistical testing; scale bar represents 100μm.

Supplementary Figure 4

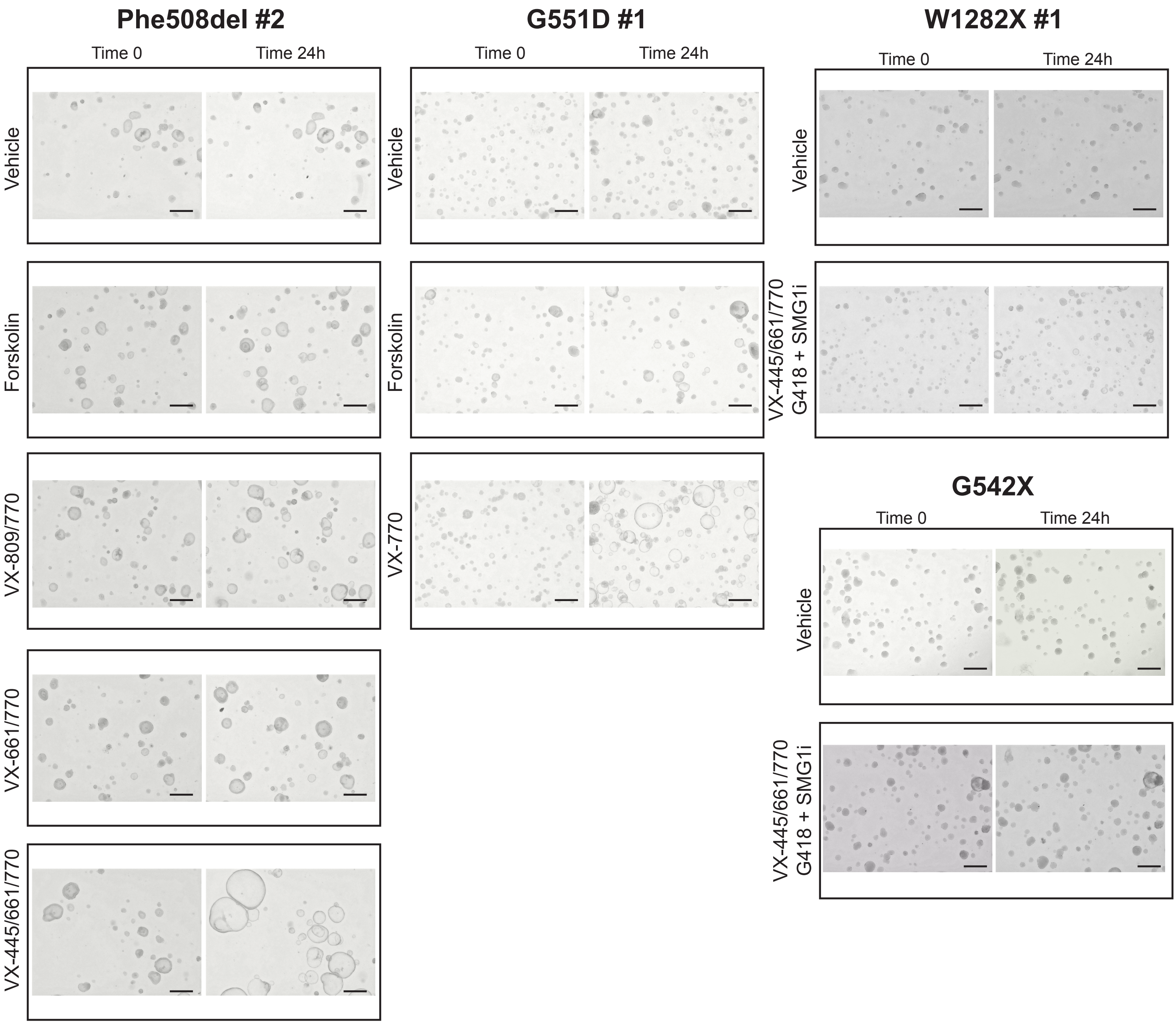

Supplementary Figure 4. Spheroid size and morphology before and after FIS. Representative images of spheroids (from n>3 biological replicates from independent differentiations) from indicated *CFTR* genotypes before and after forskolin stimulation. Spheroids were treated control DMSO, forskolin alone, or forskolin in combination with the treatments indicated. Scale bars represent 500µm.

Supplementary Figure 5

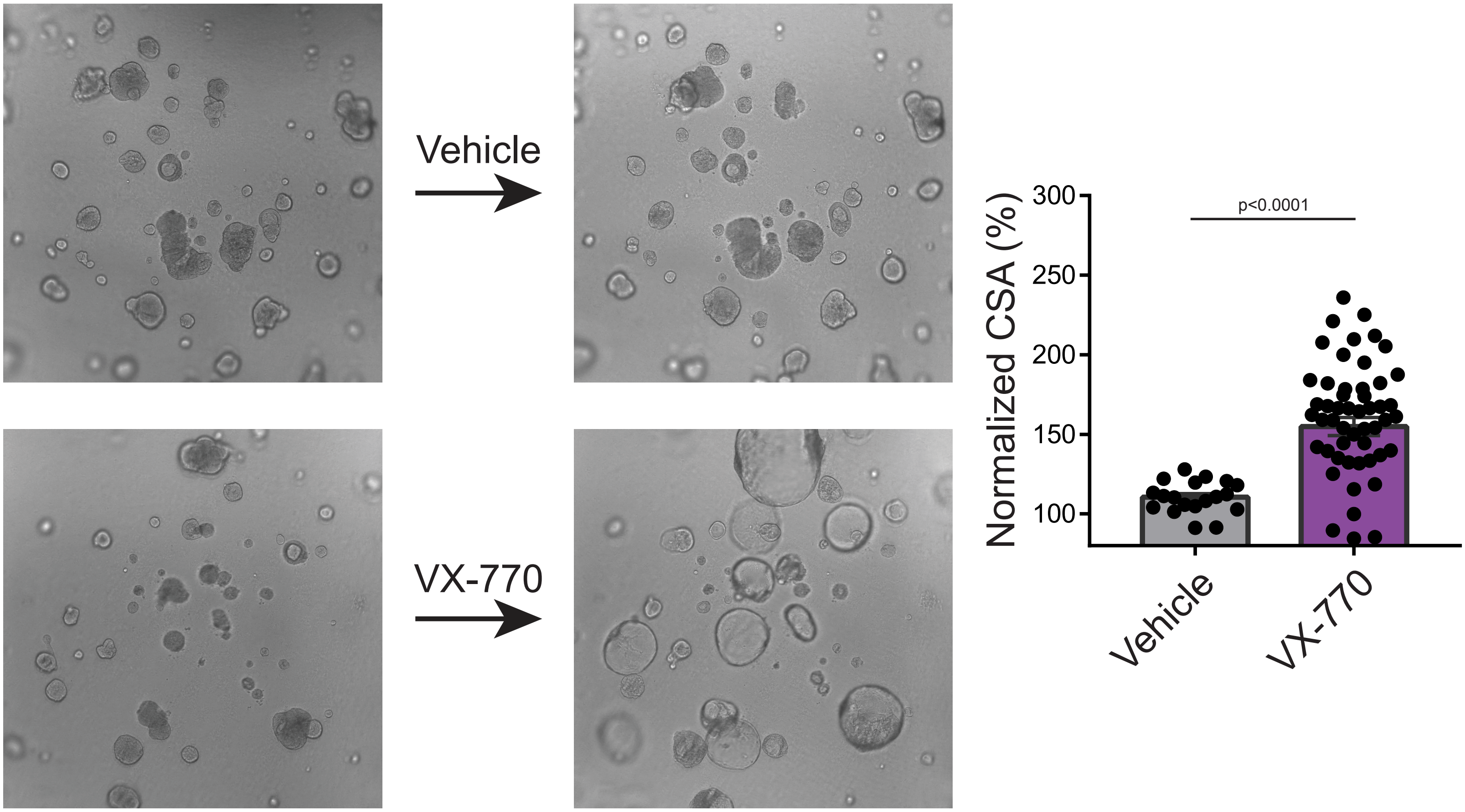

Supplementary Figure 5. iPSC-derived airway epithelial spheroids can be plated in 384-well format with demonstration of drug response. Day 30 iPSC-derived airway epithelial spheroids were generated from G551D and plated into 384-well tissue culture plates using an automated liquid handler. CSA was compared pre- and post-treatment with quantification shown at the right. Each point represents an individual sphere (n=19 for vehicle, n=54 for VX-770). P-values shown were calculated using two-tailed unpaired Student's t-test.

Supplementary Figure 6

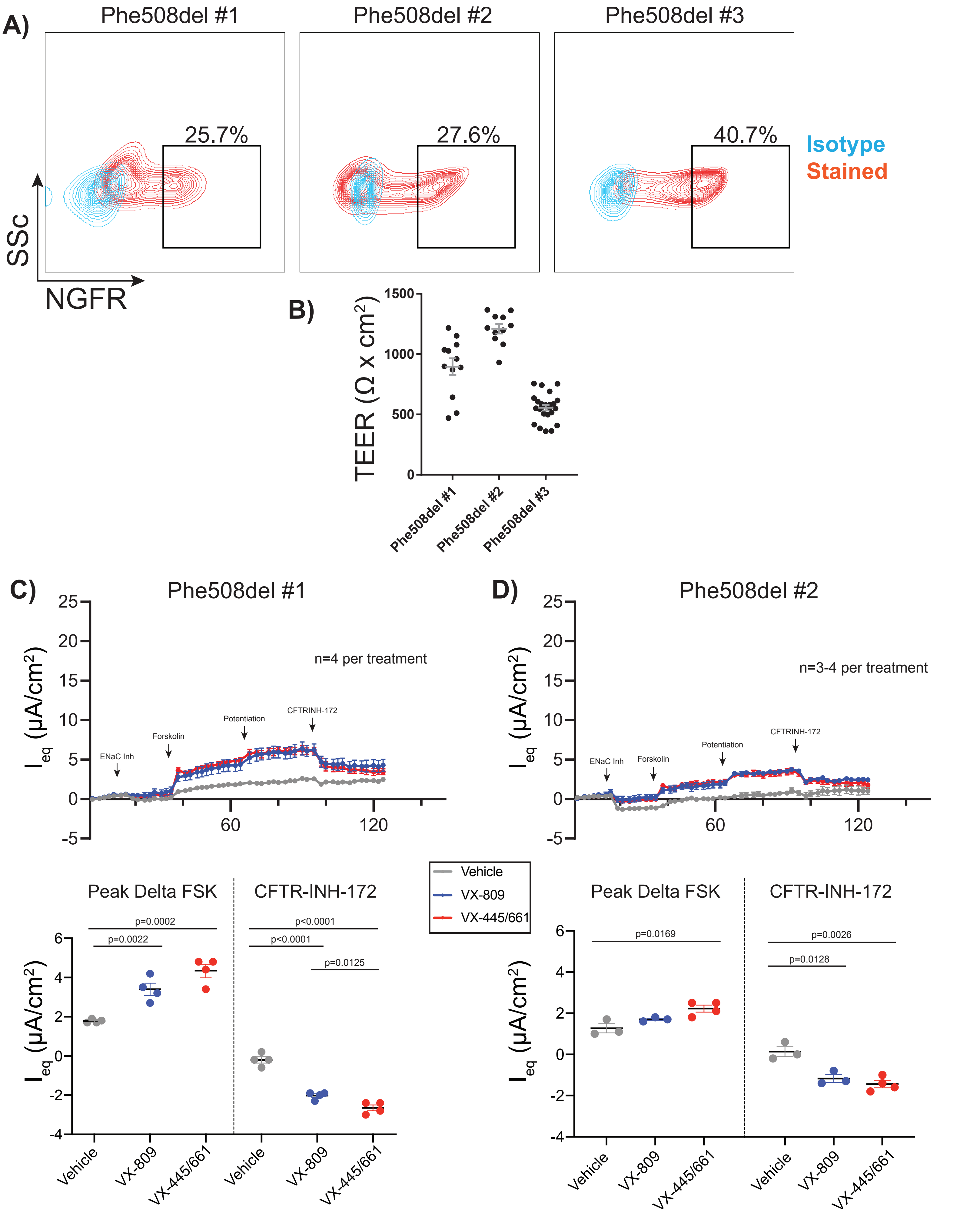

Supplementary Figure 6. Generation and equivalent current assay of Phe508del iPSC-derived mucociliary cultures. A) NGFR+ airway basal cells were identified using flow cytometry and sorted using the gating strategy shown. Stained samples are shown in red, isotype controls shown in blue. **B)** TEER of mucociliary cultures of Phe508del #1-3. Each point indicates an individual transwell insert. **C-D)** Compiled plots from equivalent current assays of Phe508del #1 and #2. Cultures were pre-treated with either DMSO (grey), VX-809 (blue), or VX-445/661 (red) prior to electrophysiologic assessment. Shown at bottom panel is the quantification of peak forskolin and CFTR-inhibitor effects. Points and error bars represent mean and standard error (n=4 experimental replicates from independent wells of a differentiation, except n=3 for Phe508del #2 vehicle and VX-809). P-values shown were calculated using unpaired Student's t-test.

Supplementary Figure 7

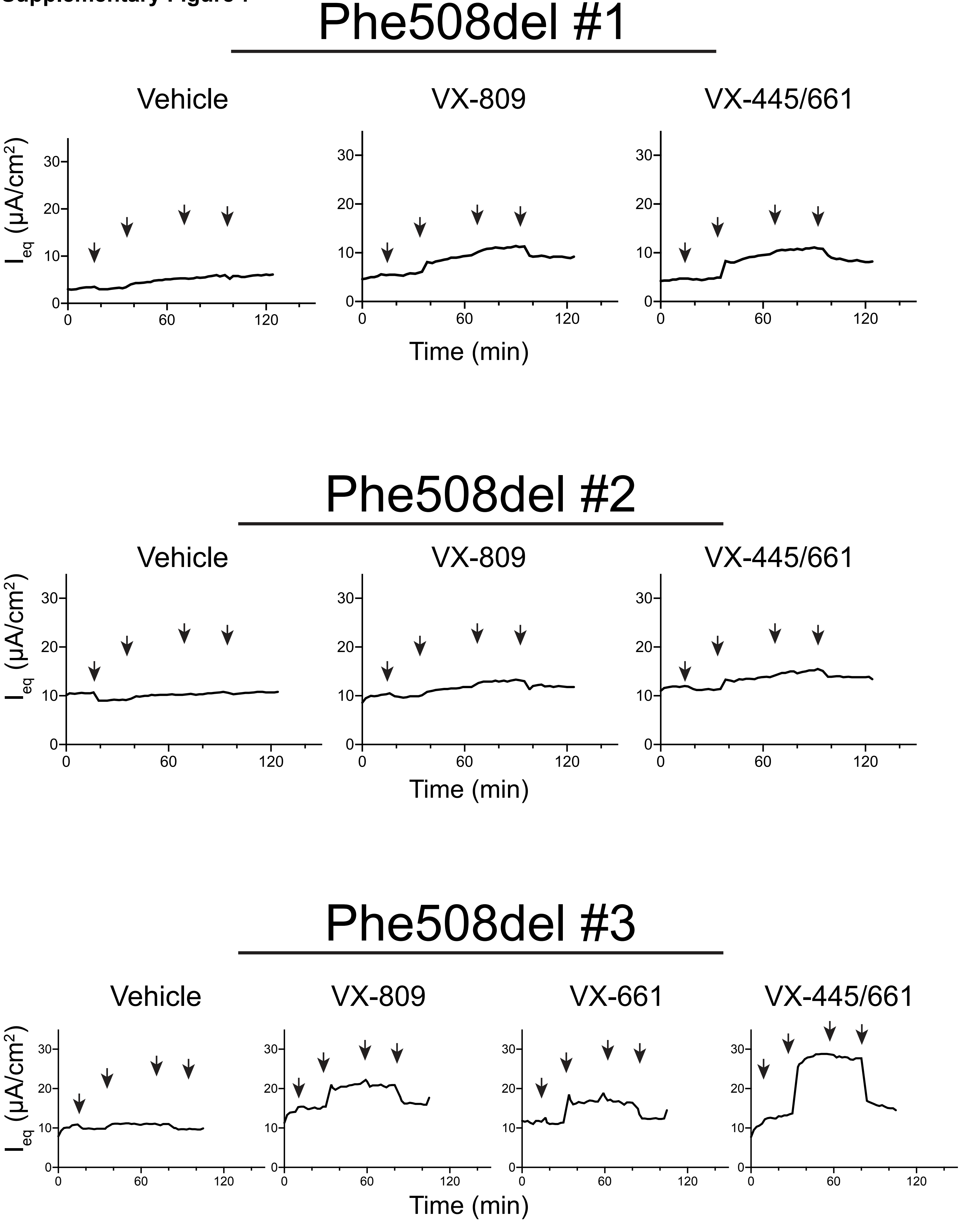

Supplementary Figure 7. Representative raw equivalent current plots for Phe508del cell lines. With the pre-treatments indicated, mucociliary cultures underwent electrophysiologic assessment. Four arrowheads shown indicate treatment with 1) Benzamil, 2) Forskolin, 3) Genistein, and 4) $CFTR_{INH}$-172.

Supplementary Figure 8

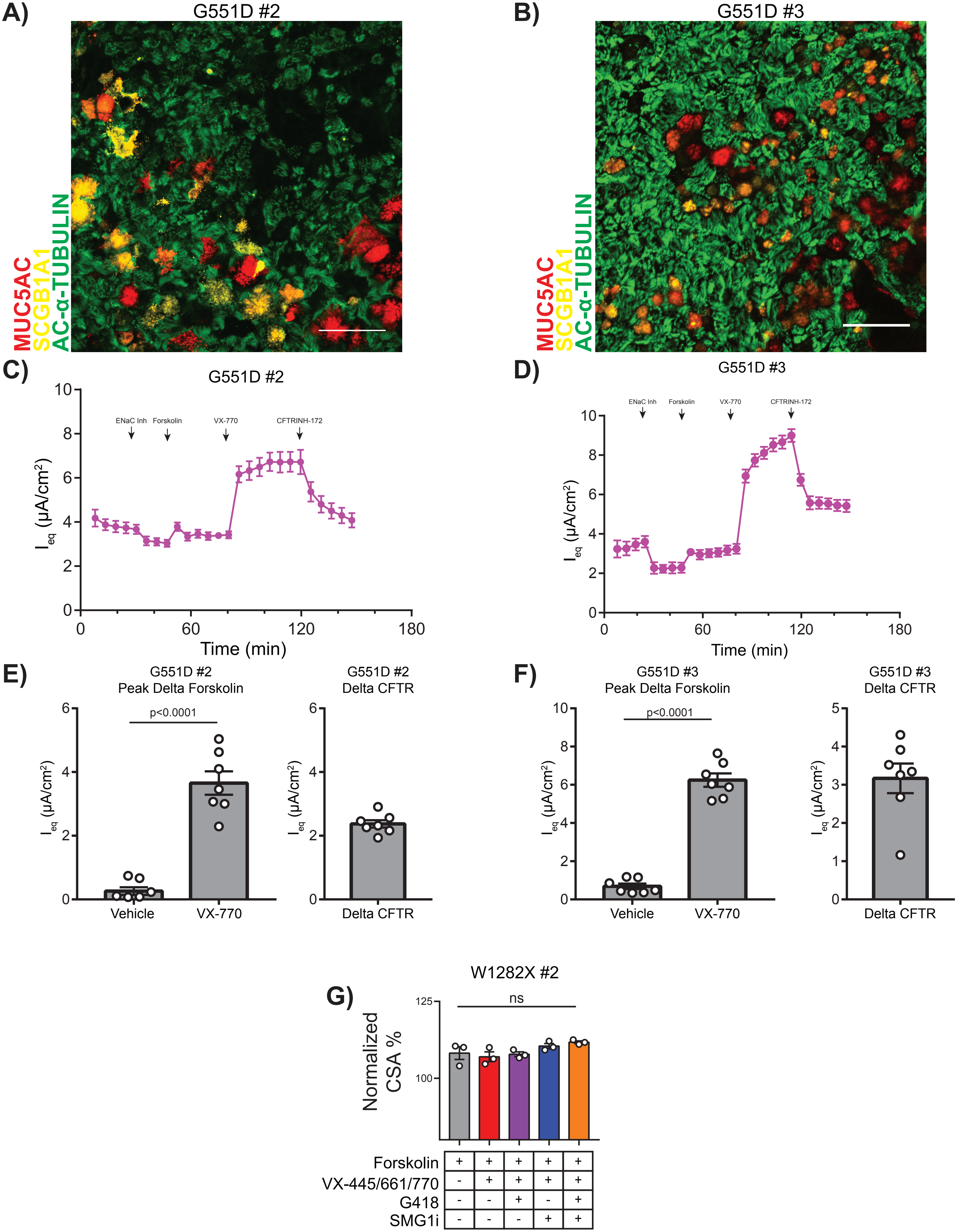

Supplementary Figure 8. G551D iPSC-derived mucociliary electrophysiologic results. A-B) Examples of immunolabeling of iPSC-derived G551D ALI cultures with antibodies against markers of multiciliated (acetylated-α-Tubulin), mucus secreting (MUC5AC), and Club (SCGB1A1) cells. **C-F)** Electrophysiologic assessment (C-D) and quantification (E-F) of equivalent current responses of G551D iPSC-derived mucociliary cultures with vehicle versus VX-770 treatment (n=7 experimental replicates from independent wells of a differentiation, except n=6 for G551D #2 vehicle). **G)** W1282X #2 FIS response after treatment combinations shown (n=3 biological replicates from independent differentiations). P-values were calculated using unpaired Student's t-test. Scale bars represent 50μm. Lines and error bars represent mean ± standard error.

Supplementary Figure 9

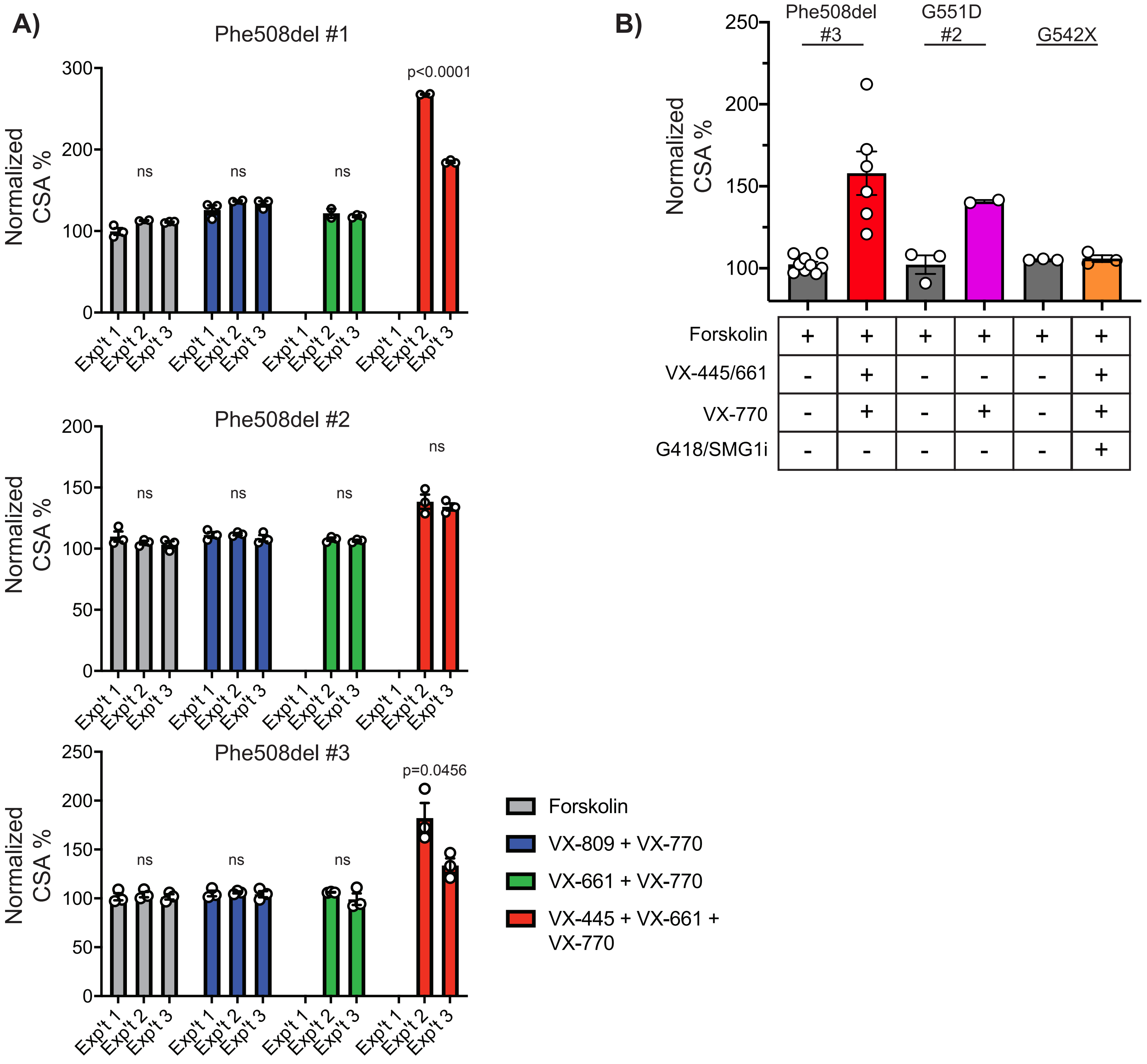

Supplementary Figure 9. Inter-experiment variability and effect of gene-editing. A) Each graph shows the FIS response for the donor specified, organized by drug treatment and experiment. Each experiment shown (1-3) was performed at a different calendar date, with a different batch of culture medium and CFTR modulator, and was separated by several months (n=3 biological replicates from independent differentiations, except n=2 for Phe508del #1 experiment 2). **B)** Effect of gene-editing of the CFTR locus in the "C17" genetic background. Conditions tested are shown on the x-axis and the indicated responses vary based on CFTR variant (n=3 biological replicates from independent differentiations, except n=9 for Phe508del #3 forskolin, n=6 for Phe508del #3 VX-445/661/770, n=2 for G551D #2 VX-770). P-values were calculated using two-tailed paired Student's t-test. Scale bars represent 50μm. Lines and error bars represent mean ± standard error.

Supplementary Table 1

Product	Company	Catalog #
2-D Matrigel	Corning	354277
mTESR1	StemCell Technologies	05850
Y-27632	Tocris	1254
Gentle Cell Dissociation Reagant	StemCell Technologies	100-0485
Dulbecco's Modified Eagle Medium/Nutrient Mixture (DMEM):F-12	ThermoFisher Scientific	11330-032
Trypsin-EDTA Solution, 0.05%	Invitrogen	T3924
Fetal Bovine Serum, ES	Fisher	SH3007003E
Definitive Endoderm Kit	StemCell Technologies	05110
Iscove's Modified Dulbecco's Medium (IMDM)	ThermoFisher Scientific	12440-053
Ham's F12	Corning	10-080-CV
Glutamax	Gibco	35050-061
1-Thioglycerol	Sigma	M6145
Bovine Serum Albumin, Fraction V (7.5% solution)	ThermoFisher Scientific	15260-037
Ascorbic acid	Sigma	A4544-25G
B27 (with retinoic acid)	Gibco	17504-044
N2 Supplement	Gibco	17502-048
Primocin	InvivoGen	ant-pm-2
Dorsomorphin	Stemgent	04-0024
SB431542	Tocris	1614
CHIR99021	Tocris	4423
Recombinant human bone BMP4 (rhBMP4)	R&D Systems	314-BP
Retinoic acid	Sigma	R2625
FGF2	R&D Systems	233-FB
FGF10	R&D Systems	345-FG-025
Dexamethasone	Sigma	D4902
8-bromoadenosine 30,50-cyclic monophosphate sodium salt (cAMP)	Sigma	B7880
3-Isobutyl-1-methylxanthine	Sigma	I5879
3-D growth factor reduced matrigel	Corning	356230
Calcein Blue, AM	Life Technologies	C1429
Dispase II, Powder	ThermoFisher Scientific	17105-041
Hanks' Balanced Salt Solution (HBSS)	ThermoFisher Scientific	14175-079
Ethylenediaminetetraacetic acid disodium salt solution (EDTA)	Sigma	E7889
HEPES	Sigma	H3375
Pneumacult Ex-Plus	StemCell Technologies	05040
DMH1	ThermoFisher Scientific	412610
A83-01	ThermoFisher Scientific	293910
Pneumacult ALI Medium	StemCell Technologies	05001
VX-809	Selleckchem	S1565
VX-661	Selleckchem	S7059
VX-445	Selleckchem	S8851
VX-770	Selleckchem	S1144
G418	Selleckchem	S3028
Forskolin	Sigma	F3917
DMSO	Sigma	D8418
Dulbecco's Phosphate Buffered Saline (1X)	Gibco	14190-144
Normal Donkey Serum	Sigma	566460
Triton X-100	Sigma	9002-93-1
QIAzol Lysis Reagant	QIAGEN	79306
TaqMan Fast Universal PCR Master Mix (2X), no AmpErase UNG	ThermoFisher Scientific	4364103
StemFlex medium	ThermoFisher Scientific	A3349401
Hoescht 33342, trihydrochloride, trihydrate	Invitrogen	H3570
ProLong Gold antifade reagant	Invitrogen	P36930

Antibody	Dilution used	Company	Catalog #
Mouse monoclonal anti-c-Kit, APC-conjugated	1:100 (Flow)	ThermoFisher Scientific	CD11705
Mouse monoclonal anti-human CD184 (CXCR4), PE-conjugated	1:100 (Flow)	StemCell Technologies	60089PE
PerCP/Cy5.5-mouse monoclonal anti-CD47	1:100 (Flow)	Biolegend	323123
PE-mouse monoclonal anti-CD26	1:100 (Flow)	Biolegend	302705
Rabbit monoclonal anti-TTF1	1:500 (Flow); 1:300 (IF)	Abcam	76013
Mouse monoclonal anti-human CD271/NGFR, APC-conjugated	1:100 (Flow)	Biolegend	345108
Rabbit monoclonal anti-Acetylated Tubulin (ACT)	1:100 (IF)	Millipore-Sigma	T7451
Rabbit monoclonal anti-MUC5AC	1:100 (IF)	Cell Signaling Technologies	61193
Mouse monoclonal anti-TP63	1:100 (IF)	Biocare	CM163A
Chicken polyclonal anti-KRT5	1:1000 (IF)	Biolegend	905901
Rabbit monoclonal anti-human SCGB3A2	1:50 (IF)	Abcam	ab181853
Mouse monoclonal anti-NGFR	1:100 (IF)	Invitrogen	MA5-13314
Donkey anti-rabbit Alexa 488	1:500	Jackson Labs	711-545-152
Donkey anti-mouse Alexa 647	1:500	Jackson Labs	715-605-150
Donkey anti-mouse Cyanine Cy5	1:500	Jackson Labs	715-175-150
Donkey anti-chicken Alexa 488	1:500	Jackson Labs	703-545-155
Mouse IgG1kappa isotype control, APC-conjugated	1:100 (Flow)	Biolegend	400122
Mouse IgG1 isotype control, PE-conjugated	1:100 (Flow)	Biolegend	400113
Mouse IgG1 isotype control, PerCP/Cy5.5-conjugated	1:100 (Flow)	Biolegend	400149

Kit	Company	Catalog #
RT kit	Applied Biosystems	98080234
RNA extraction kit	Qiagen	74014

Taqman probe	Gene	Company	Catalog #
Hs01565544_m1	CFTR	ThermoFisher Scientific	4351372
Hs00968940_m1	NKX2.1	ThermoFisher Scientific	4331182
Hs00369678_m1	Scgb3a2	ThermoFisher Scientific	4331182
Hs00170192_m1	Scgb1a1	ThermoFisher Scientific	4331182
Hs00873651_m1	Muc5AC	ThermoFisher Scientific	4331182
Hs00230964_m1	FoxJ1	ThermoFisher Scientific	4331182
Hs00978340_m1	TP63	ThermoFisher Scientific	4331182

Supplementary Table 2

<table>
<tr><th>Edit</th><th>Guide RNA (targeting portion)</th><th>Sequence of ssODN HDR donor template</th><th>Sequencing primers</th></tr>
<tr><td>G551 (WT) --> G551D</td><td>5’-GGTGGAATCACACTGAGTGG -3’</td><td>5’-CATGAATGACATTTACAGCAAATGCTTGCTAGACCAATAATTAGTTATTCACCTTGCTAAAGAAATTCTTGCTCGTTGATCTCCACTCAGTGTGATTCCACCTTCTCCAAGAACTATATTGTCTTTC -3’</td><td>5’- CATGGAAGCCCAGTGAAGAT -3’
5’- CCAAGATACGGGCACAGATT -3’</td></tr>
<tr><td>I507del --> WT</td><td>5’-ACCATTAAAGAAAATATCTT-3’</td><td rowspan="2">5’-ATAGGAAACACCAAAGATGATATTTTCTTTAATGGTGCCAGGCATAATCCAGGAAAACTGAGAACAGAATGAAATTCTTCCACTGTG-3’
5’-GCTTCTGTATCTATATTCATCATAGGAAACACCAAAGATGATATTTTCTTTAATGGTGCCAGGCATAATCCAGGAAAACTGAGAACAGAATGAAATTCTTCCACTGTG-3’
5’- GTTTCTTACCTCTTCTAGTTGGCATGCTTTGATGACGCTTCTGTATCTATATTCATCATAGGAAACACCAAAGATGATATTTTCTTTAATGGTG-3’</td><td>5’- CTGAATCATGTGCCCCTTCT -3’
5’- TCTCTGCTGGCAGATCAATG -3’</td></tr>
<tr><td>F508del --> WT</td><td>5’-ACCATTAAAGAAAATATCAT-3’</td><td>5’- CTGAATCATGTGCCCCTTCT -3’
5’- TCTCTGCTGGCAGATCAATG -3’</td></tr>
</table>

Supplementary Table 3

Guides used (targeting portion) 5' -> 3'	Screening primers 5' → 3'
ACAGCGCATCAAAGAGGAGG<br>AACAGCGCATCAAAGAGGAG<br>CAACAGCGCATCAAAGAGGA<br>TAAGCAACAGCGCATCAAAG	ATTGGGCATCATCACTTCCT<br>CGCATCAAAGAAGAAGGAGAGA
