# Supplementary material for: A multimodal iPSC platform for cystic fibrosis drug testing
Source: Nat Commun. 2022 Jul 29;13:4270. doi: 10.1038/s41467-022-31854-8 (PMC9338271; doi:10.1038/s41467-022-31854-8)
Supplement: Supplementary file 3 — Description of Additional Supplementary Files [file 41467_2022_31854_MOESM3_ESM.pdf]

File name: Supplementary Movie 1

Description: Forskolin Induced Swelling. Time-lapse microscopy depicting forskolin induced swelling of non-CF airway epithelial spheroids. Day 30 airway spheroids were stimulated with forskolin and imaged (at 4x magnification) every 15 minutes for 24 hours using a Keyence BZ-X700 fluorescence microscope. Images were stitched together and are shown at an increased rate of speed.

File name: Supplementary Movie 2

Description: Mucus Hurricane. Motile cilia and mucus hurricane within iPSC-derived ALI culture. Phe508del #2 live mucociliary cultures were imaged (at 4x magnification) using a Keyence BZ-X700 fluorescence microscope 14 days after air-exposure. There are abundant beating motile cilia in all visible fields of the culture as well as a slowly rotating (clockwise) 'mucus hurricane,' features seen also in primary HBEC ALI cultures.
