# Supplementary material for: A multimodal iPSC platform for cystic fibrosis drug testing
Source: Nat Commun. 2022 Jul 29;13:4270. doi: 10.1038/s41467-022-31854-8 (PMC9338271; doi:10.1038/s41467-022-31854-8)
Supplement: Supplementary file 6 — Reporting Summary [file 41467_2022_31854_MOESM6_ESM.pdf]

nature portfolio

Corresponding author(s): Finn Hawkins

Last updated by author(s): Jun 6, 2022

Reporting Summary

Nature Portfolio wishes to improve the reproducibility of the work that we publish. This form provides structure for consistency and transparency in reporting. For further information on Nature Portfolio policies, see our Editorial Policies and the Editorial Policy Checklist.

Statistics

For all statistical analyses, confirm that the following items are present in the figure legend, table legend, main text, or Methods section.

n/a	Confirmed	
☐	☒	The exact sample size (*n*) for each experimental group/condition, given as a discrete number and unit of measurement
☐	☒	A statement on whether measurements were taken from distinct samples or whether the same sample was measured repeatedly
☐	☒	The statistical test(s) used AND whether they are one- or two-sided<br>*Only common tests should be described solely by name; describe more complex techniques in the Methods section.*
☐	☒	A description of all covariates tested
☐	☒	A description of any assumptions or corrections, such as tests of normality and adjustment for multiple comparisons
☐	☒	A full description of the statistical parameters including central tendency (e.g. means) or other basic estimates (e.g. regression coefficient) AND variation (e.g. standard deviation) or associated estimates of uncertainty (e.g. confidence intervals)
☐	☒	For null hypothesis testing, the test statistic (e.g. *F*, *t*, *r*) with confidence intervals, effect sizes, degrees of freedom and *P* value noted<br>*Give P values as exact values whenever suitable.*
☐	☒	For Bayesian analysis, information on the choice of priors and Markov chain Monte Carlo settings
☐	☒	For hierarchical and complex designs, identification of the appropriate level for tests and full reporting of outcomes
☒	☐	Estimates of effect sizes (e.g. Cohen's *d*, Pearson's *r*), indicating how they were calculated

Our web collection on statistics for biologists contains articles on many of the points above.

Software and code

Policy information about availability of computer code

Data collection	For the scRNAseq data presented here, the sample was processed using the standard Cell ranger v.3 pipeline (Hawkins et al., 2021).
Data analysis	Automated analysis of the forskolin induced swelling (FIS) assay of airway epithelial spheroids was performed using Organoseg, a program that has been validated, used, and published elsewhere (Borten et al., 20 18). As previously described the sc- RNAseq data presented here was performed with downstream analysis and QC done on Seurat; differential expression using MAST models (Hawkins et al., 2021).

For manuscripts utilizing custom algorithms or software that are central to the research but not yet described in published literature, software must be made available to editors and reviewers. We strongly encourage code deposition in a community repository (e.g. GitHub). See the Nature Portfolio guidelines for submitting code & software for further information.

Data

Policy information about availability of data

All manuscripts must include a data availability statement. This statement should provide the following information, where applicable:

- Accession codes, unique identifiers, or web links for publicly available datasets
- A description of any restrictions on data availability
- For clinical datasets or third party data, please ensure that the statement adheres to our policy

The data presented in this manuscript are available from the authors upon reasonable request. Single- cell RNA sequencing data accession: Series GSE142246.

Human research participants

Policy information about studies involving human research participants and Sex and Gender in Research.

Reporting on sex and gender	*Use the terms sex (biological attribute) and gender (shaped by social and cultural circumstances) carefully in order to avoid confusing both terms. Indicate if findings apply to only one sex or gender; describe whether sex and gender were considered in study design whether sex and/or gender was determined based on self-reporting or assigned and methods used. Provide in the source data disaggregated sex and gender data where this information has been collected, and consent has been obtained for sharing of individual-level data; provide overall numbers in this Reporting Summary. Please state if this information has not been collected. Report sex- and gender-based analyses where performed, justify reasons for lack of sex- and gender-based analysis.*
Population characteristics	*Describe the covariate-relevant population characteristics of the human research participants (e.g. age, genotypic information, past and current diagnosis and treatment categories). If you filled out the behavioural & social sciences study design questions and have nothing to add here, write "See above."*
Recruitment	*Describe how participants were recruited. Outline any potential self-selection bias or other biases that may be present and how these are likely to impact results.*
Ethics oversight	*Identify the organization(s) that approved the study protocol.*

Note that full information on the approval of the study protocol must also be provided in the manuscript.

Field-specific reporting

Please select the one below that is the best fit for your research. If you are not sure, read the appropriate sections before making your selection.

☒ Life sciences ☐ Behavioural & social sciences ☐ Ecological, evolutionary & environmental sciences

For a reference copy of the document with all sections, see nature.com/documents/nr-reporting-summary-flat.pdf

Life sciences study design

All studies must disclose on these points even when the disclosure is negative.

Sample size	We have included twelve separate pluripotent stem cell lines in this manuscript, which have originated from nine distinct individuals (genetic backgrounds). As reprogrammed iPSCs from specific patients are not widely available, we worked to identify and utilize three donor samples from each CFTR class (1, 2, 3) and unaffected (non-CF) donors as well.
Data exclusions	No data were excluded from analysis. To ensure reproducibility of our findings we developed a protocol with several quality control check points and only iPSC differentiations that passed our quality control metrics for efficient lung progenitor specification and subsequent high quality airway differentiation were included in the electro-physiological assessments of CFTR function.
Replication	For all included mRNA expression data (qPCR) and forskolin induced swelling data, each cell line underwent a minimum of three independent directed differentiations, which were kept as separate biological experiments throughout. These are represented as individual points on the representative figures and indicated in the legends. For both qPCR and FIS, samples were also performed in technical triplicate. For 2D ALI culture electrophysiology, one distinct biological replicate is presented for each cell line. Electrophysiology conditions were carried out in at least three individual wells per treatment condition as indicated in the figures and accompanying legends.
Randomization	This is not relevant to our study.
Blinding	Blinding was not performed in our study.

Reporting for specific materials, systems and methods

We require information from authors about some types of materials, experimental systems and methods used in many studies. Here, indicate whether each material, system or method listed is relevant to your study. If you are not sure if a list item applies to your research, read the appropriate section before selecting a response.

Materials & experimental systems

n/a	Involved in the study
☐	☒ Antibodies
☐	☒ Eukaryotic cell lines
☒	☐ Palaeontology and archaeology
☒	☐ Animals and other organisms
☒	☐ Clinical data
☒	☐ Dual use research of concern

Methods

n/a	Involved in the study
☒	☐ ChIP-seq
☐	☒ Flow cytometry
☒	☐ MRI-based neuroimaging

Antibodies

Antibodies used

Mouse monoclonal anti-c-Kit, APC-conjugated, (ThermoFisher Scientific), (CD11705)
Mouse monoclonal anti-human CD184 (CXCR4), PE-conjugated, (StemCell Technologies), (60089PE)
Mouse monoclonal anti-human CD47-PerCP/Cy5.5 (Biolegend B191878)
PE-mouse monoclonal anti-CD26, (Biolegend), (302706)
Rabbit monoclonal anti-TTF1 (Abcam), (76013)
Mouse monoclonal anti-human CD271/NGFR, APC-conjugated, (Biolegend), (345108)
Rabbit monoclonal anti-Acetylated Tubulin (ACT), (Millipore-Sigma), (T7451)
Rabbit monoclonal anti-MUC5AC, (Cell Signaling Technologies), (61193)
Mouse monoclonal anti-TP63, (Biocare), (CM163A)
Chicken polyclonal anti-KRT5, (Biolegend), (905901)
Rabbit monoclonal anti-human SCGB3A2, (Abeam), (ab181853)
Donkey anti-rabbit Alexa 488, (Jackson Labs), (711-545-152)
Donkey anti-mouse Alexa 647, (Jackson Labs), (715-605-150)
Donkey anti-mouse Cyanine Cy5, (Jackson Labs), (715-175-150)
Donkey anti-chicken Alexa 488, (Jackson Labs), (703-545-155)
Mouse lgGlkappa isotype control, APC-conjugated, (Biolegend), (400122)
Mouse lgGI isotype control, PE-conjugated, (Biolegend), (400113)
Mouse lgGI isotype control, PerCP/Cy5.5-conjugated, (Biolegend), (400149)

Validation

Isotypes (or confirmed negatively expressing cell types) were used as depicted in the manuscript and figures to control for non-specific binding of primary antibodies.

Eukaryotic cell lines

Policy information about cell lines and Sex and Gender in Research

Cell line source(s)

As shown in Supplementary Figure 1, the sources of cell lines were embryonic stem cells, dermal fibroblasts, or PBMCs.

Authentication

Cell lines were all confirmed to be pluripotent by expression of traditional protein markers of pluripotency and were karyotypically normal, both as shown in Supplementary Figure l.

Mycoplasma contamination

All samples were confirmed to be negative for mycoplasma.

Commonly misidentified lines
(See ICLAC register)

None

Flow Cytometry

Plots

Confirm that:

☒ The axis labels state the marker and fluorochrome used (e.g. CD4-FITC).

☒ The axis scales are clearly visible. Include numbers along axes only for bottom left plot of group (a 'group' is an analysis of identical markers).

☒ All plots are contour plots with outliers or pseudocolor plots.

☒ A numerical value for number of cells or percentage (with statistics) is provided.

Methodology

Sample preparation

Cells were processed and prepared as previously described and indicated in the Methods section of this manuscript. Upon cell harvesting, cells were filtered to achieve a single cell suspension and then counted prior to staining. Dependent on antibody target type (i.e., cytoplasmic/nuclear vs cell surface), cells were either fixed and permeabilized or stained with primary antibody immediately (with dilutions shown in supplemental table 1). After 30 minutes of primary antibody staining, cells were washed, then stained with a secondary antibody (shown in supplemental table 1) for 30 minutes (protected from

light). Finally, cells were washed again and filtered prior to cytometric analysis. For live (non-fixed cells), a cell viability dye (e.g., Calcein Blue) was added.

Instrument	Two flow cytometers were employed for this study - BD FACSCaliber or Stratedigm SIO00EON.
Software	Flow cytometry samples were analyzed using FlowJo v 10.7.
Cell population abundance	For the day 15 (CD47 /26) cell sorting, post-sort enrichment was documented in Supplementary Figure 2 (column 4). Briefly, post-sort samples were fixed, permeabilized, and stained for intracellular NKX2-1 to confirm enrichment for NKX2-1 in the CD47hi/CD26neg sorted cells.
Gating strategy	All flow cytometry plots shown in this manuscript were gated first for non-fragments (SSc/FSc), singlets (FSc-LinH/FSc/LinA), and live (calcein blue AM positive) cells.

☒ Tick this box to confirm that a figure exemplifying the gating strategy is provided in the Supplementary Information.
